# A rapid PCR-free next-generation sequencing method for comprehensive diagnosis of chromosome disease syndromes in prenatal samples

**DOI:** 10.1097/MD.0000000000037610

**Published:** 2024-03-29

**Authors:** Hong Su, Shengni Liu, Hongxia Xu, Cuihua Shen, Min Xu, Jing Zhang, Dongyun Li

**Affiliations:** a Department of Obstetrics, Kunming Maternal and Child Care Hospital, Kunming, Yunnan, China; b BSc(Hons) Biomedical Science, University of Bristol, Bristol, England; c Department of Reproductive Medicine, The First People’s Hospital of Yunnan Province, The Affiliated Hospital of Kunming University of Science and Technology, Kunming, Yunnan, China, National Health Commission Key Laboratory of Preconception Health Birth in Western China, Kunming, Yunnan, China.

**Keywords:** chromosomal diseases, high, rCNV, risk pregnancy, seq

## Abstract

The aim of this study is to investigate the application performance of rapid copy number variation sequencing (rCNV-seq) technology for the detection of chromosomal abnormalities during prenatal diagnosis. Samples were collected from 424 pregnant women who were at high-risk for noninvasive prenatal screening in Kunming Maternal and Child Care Hospital from January 2018 to May 2022. rCNV-seq technique was used to detect fetal chromosome abnormalities and compare the results with that of chromosomal karyotype analysis. The Result showed that 330 (77.83%, 330/424) cases indicated chromosomal abnormalities among 424 high-risk pregnant women who underwent rCNV-seq. Moreover, 94 (22.17%, 94/424) cases were discovered to have copy number variations. Among the 330 fetuses with chromosomal abnormalities, common autosomal aneuploidy was observed in 203 cases (47.87%, 203/424) and sex chromosome aneuploidy was observed in 91 cases (21.46%, 91/424). Moreover, the abnormalities in multiple chromosomes were discovered in 33 cases (7.78%, 33/424), and the rare autosomal aneuploidy was observed in 3 cases (0.71%, 3/424). There were 63 fetuses (14.86%, 63/424) with pathogenic CNVs among the 94 fetuses with variable copy numbers. Of the 245 pregnant women who voluntarily selected G-band karyotyping, 1 fetus with copy number variation had normal karyotype results, and the remaining women were consistent with rCNV-seq. Our study revealed that rCNV-seq has higher accuracy in detecting common trisomy and can also detect chromosomal microdeletions or microduplications that cannot be detected by G-banding karyotype analysis. There is no effective treatment for chromosomal diseases, so it is particularly important to prevent chromosomal diseases through genetic counseling and prenatal diagnosis of chromosomal diseases.

## 1. Introduction

Prenatal diagnosis is mainly the diagnosis of chromosomal abnormalities.^[1]^ It refers to additional confirmatory tests conducted on fetuses identified as high-risk through prenatal screening, such as those identified as high-risk by the test for Down syndrome or the noninvasive prenatal testing (NIPT), or those with a family history of genetic disorders.^[2,3]^ Current studies and reports have shown that more than 200 chromosomal diseases affect human health.^[4,5]^ Chromosome diseases often manifest as a series of clinical symptoms involving multiple organs and systems. They are also significant causes of infertility, recurrent miscarriage, stillbirth, and birth defects.^[6]^ Copy number variations (CNVs) are prevalent in the human genome. They typically refer to the increase or decrease in the copy number of DNA fragments that are more than 1 kb in length.^[7,8]^ They are mainly demonstrated as submicroscopic deletions and duplications. Pathogenic CNVs can also cause another severe genetic disease, which is genomic disorders. For this type of disease, the incidence is up to 15% to 30% in the children of low intellectual or developmental delay, with or without numerous abnormalities, due to unidentified causes. The incidence also exceeds 6% in fetuses with structural abnormalities by ultrasound test but normal chromosome karyotypes.^[9–11]^

During early pregnancy screening, invasive amniocentesis or chorionic villus sampling (CVS) is used after biochemical and ultrasound studies show potential abnormal fetal development to obtain prenatal samples for chromosome karyotype analysis and chromosome microarray analysis (CMA), which is the gold standard of prenatal diagnosis.^[12,13]^ Karyotype analysis can detect structural abnormalities such as an abnormal number of chromosomes as well as deletions, duplications, inversions, and translocations larger than 10 Mb (at least 5 Mb). Nevertheless, it has poor sensitivity for detecting minor (<5 Mb) deletions and duplications on chromosomes. Besides, it cannot identify the source of additional marker chromosomes accurately. The majority of fetuses with chromosomal microdeletion or microduplication syndromes may be missed when using only conventional karyotype analysis for prenatal diagnosis.^[10]^ CMA is the most prevalent method for identifying CNVs in smaller chromosomes.^[14]^ However, it is usually necessary to culture amniotic cells to produce enough cells for analysis during karyotype analysis and CMA. Moreover, the results are usually obtained 2 weeks later.^[15]^

Copy number variation sequencing (CNV-seq) is a low-depth whole-genome sequencing technology that expands genomic fragments using low DNA input (50–200 ng) and PCR steps.^[15,16]^ CNV-seq has an average resolution of 20 kb, allowing it to detect chromosomal microdeletions and microduplications that cannot be detected by karyotype analysis. Pathogenic CNVs larger than 100 kb in the genome and chromosomal non-whole-ploid hybrids with a fusion rate >5% can be detected using CNV-seq. This method has the advantages of high automation, a short detection cycle, a high throughput, and a high resolution. Based on current data, CNV-seq can detect an additional 5% to 15% of chromosomal abnormalities missed by karyotype analysis.^[10]^ The rapid copy number variation sequencing (rCNV-seq) used in this study is a sequencing method based on PCR-free libraries.^[17]^ The input DNA can be exposed to enzyme digestion without any additional selection steps, followed by end repair and dA (adenine) tail binding in a single step, without the need for PCR, making library preparation faster, easier, and more efficient. Previous studies have shown that rCNV-seq can reliably and accurately detect clinically significant CNVs. Therefore, this paper chooses the noninvasive rCNV-seq to test 424 high-risk pregnant women in NIPT compared to the expensive and less sensitive CMA. It also examines the CNV-seq results and compares them to the chromosome karyotype results. Moreover, it defines the type of fetal chromosomal abnormalities and assesses the clinical utility of CNV-seq in prenatal diagnosis.

## 2. Materials and methods

### 2.1. Study population and sample

**Figure 1.**
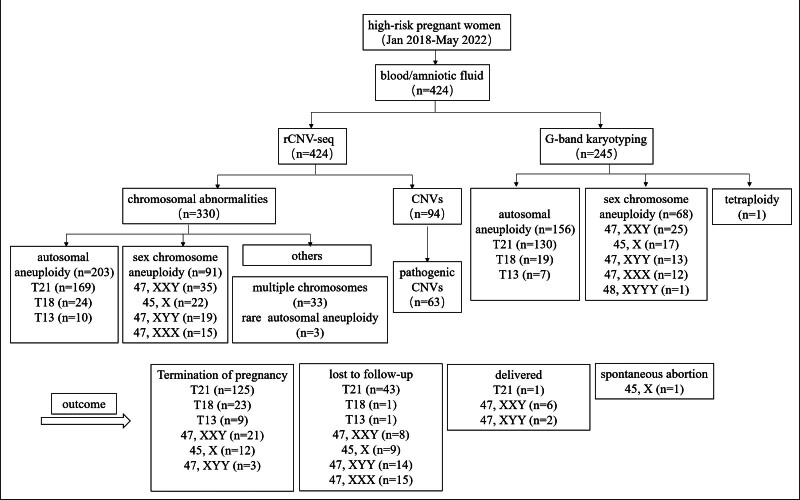
Flowchart of rCNV-seq results and clinical outcome of 424 pregnant women. rCNV-seq = rapid PCR-free next-generation sequencing.

424 pregnant women who were found to be high-risk through prenatal screening at Kunming Maternal and Child Care Hospital from January 2018 to May 2022 were enrolled as subjects. This study was approved by the Ethical Committee of Kunming Maternity and Child Health Hospital and adhered to the Helsinki Declaration: Ethical Principles for Medical Research Involving Human Subjects by World Medical Association. The inclusion criteria for all pregnant women were established based on those of the American College of Medical Genetics and Genomics^[18]^ and the National Health Commission of the People’s Republic of China (2016): Pregnant women aged ≥16 years with a singleton pregnancy ≥14 weeks, without history of blood transfusion or transplantation. A flowchart of this study is illustrated in Figure 1.

### 2.2. Copy number variation sequencing

This method is noninvasive for the fetus, It requires only 2 ml of peripheral blood from pregnant women as the experimental sample. In summary, Peripheral venous blood (2 ml) was obtained from pregnant woman. Then, genomic DNA was extracted by the TIANamp Micro DNA Kit (Tiangen Biotech). Fifity nanograms of genomic DNA was fragmented to an average size of 300 bp, and sequencing libraries were prepared as described in the literature.^[17]^

Single end sequencing was performed on the NextSeq CN500 platform (Illumina, San Diego, CA, USA) with a run time of 6.5 hours and about 5 million 45 bp raw reads were generated per sample. The Raw reads were then edited to remove the artificial adapter sequence, and the true 36 bp genome sequence was mapped to the hg19 reference genome using the Burrows and Wheeler algorithm. Averagely, approximately 2.8 to 3.2 million reads were uniquely mapped for data analysis. Reads were assigned to 20 kb bins along the length of each chromosome, and CNVs were identified from 24 chromosome copy number (CN) plots. Duplications were defined as CN > 2.8, deletions CN < 1.2, disomy (1.8 < CN < 2.2), mosaic trisomy (2.2 < CN < 2.8), and mosaic monosomy (1.2 < CN < 1.8).

### 2.3. Chromosome karyotype analysis

Pregnant women with NIPT positive results voluntarily consented to undergo an invasive prenatal diagnosis. Karyotyping was processed on the cells cultured from the trophoblastic cells or the fetal cells using a conventional Giemsa banding (G-binding) method.^[18]^

### 2.4. Pregnancy outcome follow-up

Clinical investigators reviewed neonatal outcomes based on medical records and telephone interviews. Pregnancy outcomes were recorded and classified as continued gestation, live birth, miscarriage, termination of pregnancy, and failure to follow-up.

## 3. Result

### 3.1. Characteristics of study population

424 pregnant women chose rCNV-seq for testing after NIPT indicated high-risk results. The age and gestational week distribution of pregnant women are shown in Table 1. The minimum age for pregnant women is 16 years, the maximum age is 47 years, and the average age is 31.65 years (SD: 6.45, range 16–47). 415 of the 424 pregnant women were in the middle stage of pregnancy (14–28 wk), accounting for 97.87% (415/421). On the other hand, the gestational weeks of the remaining 9 pregnant women were over 28 weeks, accounting for 2.21% (9/421).

**Table 1:** Age and gestational age distribution of 424 high-risk pregnant women.

	N	Mean ± SD	Median
Age (yr)	424	31.65 ± 6.45	31.5
16–24	63 (14.9%)	21.81 ± 2.18	22
25–29	102 (24.1%)	27.08 ± 1.37	27
30–34	108 (25.5%)	31.85 ± 1.41	32
35–39	97 (22.9%)	36.96 ± 1.46	37
40–44	49 (11.6%)	41.39 ± 1.32	41
≥45	5 (1.2%)	45.8 ± 1.09	45
GA at sampling (wk)	424	20.18 ± 2.57W	20W
Second-trimester	415 (97.87%)	19.95 ± 2.08W	19W
Third-trimester	9 (2.12%)	30.44 ± 2.00W	30W

W = weeks.

### 3.2. Performance of rapid copy number variation sequencing and follow-up

**Figure 2.**
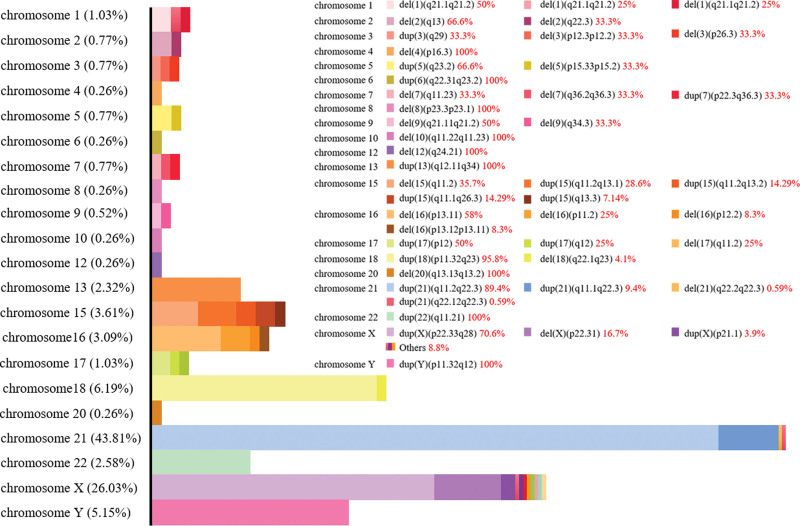
The results of chromosomal abnormalities in all pregnant women undergoing rCNV-seq. rCNV-seq = rapid PCR-free next-generation sequencing.

The results of chromosomal abnormalities in all pregnant women undergoing rCNV-seq testing are shown in Figure 2. There were 294 cases of common aneuploidies among the 424 high-risk pregnant women who voluntarily underwent rCNV-seq (Table 2), including 203 cases of trisomies 21, 18, and 13, with a detection rate of 47.87% (203/424). There were 169 cases of trisomy 21 among common aneuploidies, including 6 cases of mosaicism. Of these, 125 cases selected pregnancy termination, 1 case was delivered with normal phenotype. The main abnormal band of chromosome 21 was q11.2q22.3, accounting for 89.41%. There were 24 cases of trisomy 18, with the main abnormal region being q22.1q23, accounting for 95.83%. 23 of them selected pregnancy termination, and 1 case was lost to follow-up. 10 cases of trisomy 13, including 1 case of mosaicism. As with trisomy 13, all chose to terminate pregnancy except 1 was lost to follow-up. There were 91 fetuses tested positive for sex chromosome aneuploidy, the detection rate was 21.46% (91/424), including 35 cases (38.46%) of Klinefelter syndrome (47, XXY), and 6 cases of mosaicism. 22 of them elected pregnancy termination, 7 of them were lost to follow-up, but the other 6 decided to continue their pregnancy to have a live birth. There were 22 cases (24.18%) of Turner syndrome (45, X), and 13 were mosaicism. There was 1 case of spontaneous abortion, and all the rest chose to terminate the pregnancy. There were 19 cases (20.88%) of Jacob syndrome (47, XYY), and 3 were mosaicism. of which 3 elected pregnancy termination while the other 2 cases chose to continue the pregnancy, and the remaining 14 cases were lost to follow-up. Triple X syndrome (47, XXX) was observed in 15 cases (16.48%), all of them were lost to follow-up. Rare cases of trisomy involving autosomal chromosomes were detected in 3 cases, with a detection rate of 0.71% (3/421), located on chromosome 7 (1 case) and chromosome 15 (2 cases), and all of them chose to terminate the pregnancy. Moreover, 94 cases were detected in the detection for copy number variation, with a detection rate of 22.33% (94/421). The abnormalities in 2 or more chromosomes were detected for 32 pregnant women.

**Table 2:** Chromosome region bands, sites, fragment sizes and type of aberrations of aneuploidy.

Chromosome	Anomalous zone	Location	N
7	dup(7)(p22.3q36.3)(mos)	chr7:g.1_159138663dup	1
13	dup(13)(q12.11q34)	chr13:g.19500000_115169878dup	9
	dup(13)(q12.11q34)(mos)	chr13:g.19500000_115169878dup	1
15	dup(15)(q11.1q26.3)(mos)	chr15:g.20700000_102531392dup	2
18	dup(18)(p11.32q23)	chr18:g.1_78077248dup	24
21	dup(21)(q11.2q22.3)	chr21:g.14300000_48129895dup	147
	dup(21)(q11.1q22.3)	chr21:g.14300000_48129895dup	16
	dup(21)(q11.2q22.3)(mos)	chr21:g.14300000_48129895dup	6
X	dup(X)(p22.33q28)	chrX:g.1_155270560dup	44
	dup(X)(p22.33q28)(mos)	chrX:g.1_155270560dup	6
	del(X)(p22.33q28)	chrX:g.1_155270560del	9
	del(X)(p22.33q28)(mos)	chrX:g.1_155270560del	13
Y	dup(Y)(p11.32q12)	chrY:g.1_59373566dup	16
	dup(Y)(p11.32q12)(mos)	chrY:g.1_59373566dup	3
Total	–	–	297

mos = mosaic.

### 3.3. Karyotype analysis results

Of 424 high-risk pregnant women receiving rCNV-seq, 245 voluntarily opted for karyotype analysis. Among them, 156 cases of autosomal aneuploidies were detected, with a detection rate of 63.67% (156/245). There were 68 cases of sex chromosome aneuploidy, with a detection rate of 27.76% (68/245), and 1 case of tetraploidy, with a detection rate of 0.41% (1/245). There were 21 other abnormal cases, accounting for 8.57% of the total (21/245). Among the common aneuploidies (Table 3), trisomy 21 had 130 cases, trisomy 18 had 19 cases, and trisomy 13 had 7 cases. Among the 68 cases with sex chromosome aneuploidy (Table 4), there were 25 cases of Klinefelter syndrome (47, XXY), including 5 cases of mosaicism. There were 17 cases of Turner syndrome (45, X), including 10 cases of mosaicism. 13 cases of Jacob syndrome (47, XYY), 12 cases of triple X syndrome (47, XXX), and 1 case of Superman syndrome (48, XYYY).

**Table 3:** Karyotype analysis of autosomal aneuploidy.

Common aneuploidies	Karyotype	N
Trisomy 21	47,XY + 21	66
	47,XX + 21	53
	46,XX + 21,rob(15;22)	1
	46,XX,rob(21;21) + 21	1
	47,XX + 21,t(9;15)	1
	47,XX,inv (9) + 21	1
	47,XX,14ps+,+21	1
	46,XY,rob(15;22) + 21	1
	46,XY,rob(14;21) + 21	1
Trisomy 21 (mos)	47,XY + 21[]/46,XY[]	3
	47,XX + 21[]/46,XX[]	1
Trisomy 18	47,XX + 18	12
	47,XY + 18	6
	47,XY + 18,t(2;7)	1
Trisomy 13	47,XY + 13	3
	47,XX + 13	2
	46,XY,rob(13;13) + 13	1
Trisomy 13 (mos)	47,XX + 13[46]/46,XX[26]	1
Total	–	156

mos = mosaic.

**Table 4:** Karyotype analysis of sex chromosome aneuploidy.

Sex chromosome abnormalities	Karyotype	N
Klinefelter syndrome	47,XXY	19
	47,XXY,15ps+	1
Klinefelter syndrome (mos)	47,XXY[]/46,XY[]	5
Jacob syndrome	47,XYY	13
Superman syndrome	48,XYYY	1
Triple X syndrome	47,XXX	10
	47,XXX,1qh+,22ps+	1
	47,XXX[56]/45,X0[6]	1
Turner syndrome	45,X0	5
	45,XY,rob (15;22)	1
	46,X,+mar[14]/45,X[56]	1
Turner syndrome (mos)	45,XO[]/46,XX[]	10
Total	–	68

## 4. Discussion

Based on statistics, the incidence of birth defects in China is about 5.6%.^[19]^ Among them, chromosomal abnormalities account for over 80% of the genetic causes of birth defects,^[20]^ including abnormal chromosome numbers, large segment deletions or repeats, and pathogenic CNVs. In recent years, as a result of the implementation of the Three-child policy, the factors such as advanced maternal age and environmental issues have brought new challenges to enhancing the quality and development of the population. Therefore, it is very important to use prenatal diagnosis for prevention. CNV-seq is now widely used for prenatal diagnosis in pregnant women. Researchers have carried out a series of studies on the clinical application of this technology, fully evaluating its practicality and accuracy. According to the findings of Zhou et al,^[17]^ rCNV-seq has a high sensitivity and specificity in detecting common autosomal aneuploidies such as T21, as well as segmental aneuploidies associated with various types of whole-genome microdeletion or microduplication syndromes. Even when using as low as 10 ng of gDNA to build PCR libraries, the detection of CNVs has very high reproducibility. The rCNV-seq technology based on a PCR-free library is used in this study to perform prenatal diagnosis on 424 high-risk pregnant women samples. A total of 393 cases were detected as pathogenic, with a detection rate of 92.69%. 245 of the 424 high-risk pregnant women chose to undergo chromosomal karyotype analysis, including 242 cases with positive CNV-seq detection results and 3 pregnant women with potentially pathogenic detection results. One of these 3 pregnant women at risk of illness had normal chromosomal karyotype results, while the other 2 had chromosome 1 duplications with fragment sizes of 38.26 Mb and 7.28 Mb, respectively.

Despite the fact that CNV-seq has significantly enhanced the detection rate of genetic causes of fetal chromosomal disorders, this technology often identifies copy number changes with unclear clinical phenotype relevance in prenatal diagnosis practice, referred to as CNVs of unknown pathogenicity. In this study, the overall positive rate of unknown pathogenic CNVs is 14.86%. 24 out of 63 fetuses with pathogenic CNVs had a deletion of 0.98 Mb to 1.70 Mb on the X chromosome p22.31, accounting for 38.10% (24/63) of all pathogenic CNVs. The steroid sulphatase deficiency (STS) disease is caused by pathogenic CNVs on the X chromosome that cover the STS gene (Steroid sulfatase).^[21]^ Almost all the patients with this disease are male. It occurs after birth or shortly thereafter. The main clinical symptoms are large areas of scales on the limbs, face, neck, trunk, and buttocks, with the neck, face, and trunk being the most severely affected. Some patients may also have complications such as corneal opacity, bronchial asthma, and allergic rhinitis since skin lesions persist and do not improve with age. Some female carriers may have mild scales visible on their arms and shins. Chromosome abnormalities and pathogenic CNVs directly affect the normal growth and development of affected children. The affected children will suffer from severe structural and functional defects such as intellectual disability, multiple deformities, and reproductive disorders throughout their life, which may bring a heavy burden to their family and the society. Currently, no effective clinical treatment plan is available. Therefore, it’s very important to enhance prenatal screening and diagnosis, provide early and effective intervention for high-risk pregnant women, and reduce birth defects.

## Author contributions

**Conceptualization:** Dongyun Li.

**Data curation:** Hong Su, Cuihua Shen.

**Formal analysis:** Shengni Liu.

**Funding acquisition:** Hongxia Xu.

**Investigation:** Hong Su, Hongxia Xu, Dongyun Li.

**Methodology:** Cuihua Shen.

**Project administration:** Dongyun Li.

**Resources:** Cuihua Shen.

**Software:** Shengni Liu, Min Xu, Jing Zhang.

**Supervision:** Shengni Liu.

**Validation:** Min Xu.

**Visualization:** Min Xu, Jing Zhang.

**Writing – original draft:** Hong Su, Shengni Liu.

**Writing – review & editing:** Dongyun Li.
